# The hidden information in patient-reported outcomes and clinician-assessed outcomes: multiple sclerosis as a proof of concept of a machine learning approach

**DOI:** 10.1007/s10072-019-04093-x

**Published:** 2019-10-28

**Authors:** Giampaolo Brichetto, Margherita Monti Bragadin, Samuele Fiorini, Mario Alberto Battaglia, Giovanna Konrad, Michela Ponzio, Ludovico Pedullà, Alessandro Verri, Annalisa Barla, Andrea Tacchino

**Affiliations:** 1 Department of Research, Italian Multiple Sclerosis Foundation, Genoa, Italy; 2 AISM Rehabilitation Center of Liguria, Genoa, Italy; 3 Department of Informatics, Bioengineering, Robotics and System Engineering, University of Genoa, Genoa, Italy; 4 Department of Life Science, University of Siena, Siena, Italy

**Keywords:** Multiple sclerosis, Disease course, Disease prediction, Patient-reported outcome, Machine learning

## Abstract

Machine learning (ML) applied to patient-reported (PROs) and clinical-assessed outcomes (CAOs) could favour a more predictive and personalized medicine. Our aim was to confirm the important role of applying ML to PROs and CAOs of people with relapsing-remitting (RR) and secondary progressive (SP) form of multiple sclerosis (MS), to promptly identifying information useful to predict disease progression. For our analysis, a dataset of 3398 evaluations from 810 persons with MS (PwMS) was adopted. Three steps were provided: course classification; extraction of the most relevant predictors at the next time point; prediction if the patient will experience the transition from RR to SP at the next time point. The Current Course Assignment (CCA) step correctly assigned the current MS course with an accuracy of about 86.0%. The MS course at the next time point can be predicted using the predictors selected in CCA. PROs/CAOs Evolution Prediction (PEP) followed by Future Course Assignment (FCA) was able to foresee the course at the next time point with an accuracy of 82.6%. Our results suggest that PROs and CAOs could help the clinician decision-making in their practice.

## Introduction

Following the major revolution that is undergoing in medicine, the nature of healthcare is shifting from a disease centred to a patient-centred approach [1]. Beside emerging medical disciplines, such as genomic medicine, recently, participatory medicine is gaining increasing attention for diagnostic or therapeutic decision-making, consequently requiring new insights in patient involvement. Also, for multiple sclerosis (MS), innovative strategies to use traditional clinical measurements (i.e. patient-reported outcomes (PROs), clinician-assessed outcomes (CAOs)) and new computational tools (e.g. machine learning (ML)) could favour the shift from the current reactive medicine mode towards a personalized, predictive, preventive and participatory medicine [2]. In particular, the application of ML to PROs and CAOs could become the keystone [3] to better detect the rapid changes due to the pathology evolution and, consequently, to pave a timelier, low-cost and patient-centred way for people with MS (PwMS) management. Although, ML approaches [4] have proven to be able to extract meaningful information hidden in the data in a wide range of biomedical applications [5, 6], their role in analyzing PROs and CAOs of PwMS has still to be fully consolidated [7]. Several instrumental measures (e.g. MRI) offer established and well-known biomarkers of disease activity, especially for relapsing-remitting (RR) course of MS; those are currently less useful in detecting the transition from RR to the secondary progressive (SP) form [8]. Thus, ML applied to PROs and CAOs could be valuable to fill this gap or to improve MRI prediction power. Here, we applied previously developed machine learning algorithms [7] to a dataset (PROMOPRO-MS database) populated of PROs and CAOs exclusively from RR and SP PwMS, by confirming the decisive role that ML approaches could play in the future in timely identifying information useful to predict the progression from RR to SP course and consequently in supporting pharmacological and rehabilitative therapeutic decision-making in order to prevent this transition.

## Materials and methods

Patients followed as outpatients or at-home by Italian Multiple Sclerosis Society (AISM) Rehabilitation Centres of Genoa, Padua and Vicenza, have been progressively enrolled in the study [9] without any inclusion/exclusion criteria unless MS diagnosis. The study was approved by the local ethics committee (Comitato Etico Aziendale A.O. Universitaria “San Martino” Genova). A written informed consent was obtained from all subjects prior to study entry.

Personal (i.e. years of education), clinical (i.e. number of relapses in the last 4 months) and biometric (i.e. height and weight) data, PROs and CAOs related to the most relevant domains for MS (i.e. mobility, fatigue, cognitive performances, emotional status, bladder continence, quality of life), were acquired each 4 months from January–May 2014 to May–September 2017 for a maximum of 11 evaluations for each participant. Each sample of the dataset is represented by a vector containing 143 predictors (i.e. single question, subtotal scores and total scores) (Table 1) and adopted to finally refine previously developed ML algorithms [7].

**Table 1:** Personal, clinical, biometric, clinician assessed outcome (CAO) and patient reported outcomes (CAO) data acquired 4 months each from January-May 2014 to May-September 2017

Predictors contained in the vectors of the dataset					
	Code	Data type	No. of items	No. of subtotal scores	No. of total scores
ABILHAND	ABILH	PRO	23	-	1
Edinburgh Handedness Inventory	EHI	PRO	10	-	1
Hospital Anxiety and Depression Scale	HADS	PRO	14	2	1
Life Satisfaction Index	LSI	PRO	11	-	1
Modified Fatigue Impact Scale	MFIS	PRO	21	3	1
Overactive Bladder Questionnaire	OAB-q	PRO	8	-	1
Functional Independence Measure	FIM	CAO	19	6	1
Montreal Cognitive Assessment	MoCA	CAO	11	-	2
Paced Auditory Serial Addition Task	PASAT	CAO	-	-	1
Symbol Digit Modalities Test	SDMT	CAO	-	-	1
Education (years)	EDU	Personal	-	-	1
Relapses in the last 4 months (no.)	RELAPS	Clinical	-	-	1
Height (cm)	H	Biometric	-	-	1
Weight (kg)	W	Biometric	-	-	1
Predictors selected in disease course classification and prediction					
ABILH (item 12)	Hammering a nail				
ABILH (TOT)	ABILH total score				
HADS (item 7)	I can sit at ease and feel relaxed.				
HADS (SUB1)	HADS subtotal measuring anxiety				
HADS (SUB2)	HADS subtotal measuring depression				
HADS (TOT)	HADS total score				
LSI (TOT)	LSI total score				
MFIS (item 2)	I have had difficulty paying attention for long periods of time.				
MFIS (SUB1)	MFIS subtotal measuring cognitive status				
MFIS (SUB2)	MFIS subtotal measuring physical status				
MFIS (SUB3)	MFIS subtotal measuring psychosocial status				
MFIS (TOT)	MFIS total score				
OAB-q (item 1)	Frequent urination during the daytime hours				
OAB-q (item 4)	Accidental loss of small amounts of urine				
OAB-q (TOT)	OAB-q total score				
FIM (item 10)	How much assistance is required for toilet transfer?				
FIM (item 11)	How much assistance is required for shower transfer?				
FIM (item 12)	How much assistance is required for locomotion (ambulatory)?				
FIM (item 14)	How much assistance is required for locomotion (wheelchair)?				
FIM (SUB3)	FIM subtotal measuring sphincter control				
FIM (SUB4)	FIM subtotal measuring personal care				
FIM (SUB5)	FIM subtotal measuring locomotion				
FIM (SUB6)	FIM subtotal measuring mobility				
FIM (TOT)	FIM total score				
MoCA (item 1)	Visuoconstructional test				
MoCA (item 9)	Memory test				
MoCA (TOT1)	MOCA total score				
MoCA (TOT2)	MOCA total score corrected for year of education				
PASAT	Score				
SDMT	Score				
EDU	Years of formal education				
H	Height (cm)				
W	Weight (kg)				

We proceeded through a three-step strategy based on supervised ML algorithms in order to develop a MS evolution temporal model (i.e. from RR to SP): Current Course Assignment (CCA), PROs/CAOs Evolution Prediction (PEP) and Future Course Assignment (FCA). In particular, CCA solves a binary classification problem: each predictor vector, independently of evaluations time course, is associated with the most probable MS form (RR or SP). PEP provides an updated historical representation: for each patient, the most probable predictor vector at the next time point is predicted. FCA model is just the function composition of FCA and PEP. Therefore, it allows foreseeing if the patient at the next time point will be RR or SP, by allowing inferring if he/she will experience the transition.

## Results

A total of 3398 evaluations from 810 PwMS represented the dataset adopted for our analysis. Among those, 1451 were RR PwMS at the evaluation time and 1947 SP PwMS. The CCA step correctly assigned the current MS course with an accuracy of about 86.0%. This was obtained through a reduced number of predictors from the considered PROs and CAOs and from biometric and personal data (Table 1). Specifically, self- and physician-reported information of the physical domain (upper limb abilities, fatigue, personal care and locomotion) and few self-reported information of present and past mood and quality of life, as well as clinically assessed performances in cognitive tests (memory, calculation, information processing), were necessary.

Although important, the most interesting result was obtained from the second and third steps of the analysis. By using the predictors selected in CCA, PEP followed by FCA was able to foresee the course at the next time point with an accuracy of 82.6%.

The results suggest that the disease course prediction based on PROs and CAOs is feasible in MS. However, although a reduced number of predictors is selected with respect to those originally considered, disease course prediction at the next time point requires to acquire all 143 predictors.

## Conclusion

Here, we presented a data analysis pipeline based on ML methods that could address MS diagnosis and prognosis issues. Our results show that PROs and CAOs could be used to build accurate models of MS disease course prediction. PROs and CAOs, alone or integrated with other indexes such as MRI outcomes and biomarkers, could help the decision-making process of clinicians in their daily practice. The main limitation of the current prediction algorithm is the considerable amount of data needed. Future developments of the algorithm that will integrate also data on therapies followed by the patients already present in the current database and eventually MRI outcomes will take into account the necessity to optimize and minimize the necessary data. In conclusion, the possibility to better define the clinical complexity levels of the patient and to have sufficient and adequate predictive criteria for MS evolution could be an instrument for the construction of a more fruitful therapeutic pact between clinician and patient based on better perspective knowledge, increased disease consciousness and engagement in pharmacological and rehabilitative treatments, and physical activity, to date, is considered the main way to delay MS progression and to improve quality of life [10].
